# Low-pressure micro-mechanical re-adaptation device sustainably and effectively improves locomotor recovery from complete spinal cord injury

**DOI:** 10.1038/s42003-018-0210-8

**Published:** 2018-11-26

**Authors:** Veronica Estrada, Julia Krebbers, Christian Voss, Nicole Brazda, Heinrich Blazyca, Jennifer Illgen, Klaus Seide, Christian Jürgens, Jörg Müller, Rudolf Martini, Hoc Khiem Trieu, Hans Werner Müller

**Affiliations:** 1 Molecular Neurobiology Laboratory, Department of Neurology, Heinrich-Heine-University Medical Centre Düsseldorf, Moorenstr. 5, 40225 Düsseldorf, Germany; 2 Institute of Microsystems Technology, Hamburg University of Technology, Eißendorfer Str. 42, 21073 Hamburg, Germany; 3 BG Trauma Centre Hamburg, Bergedorfer Str. 10, 21033 Hamburg, Germany; 4 Developmental Neurobiology, Department of Neurology, University Hospital Würzburg, Josef-Schneider-Str. 11, 97080 Würzburg, Germany; 5 CNR (Center for Neuronal Regeneration), Merowinger Platz 1a, 40225 Düsseldorf, Germany; 6 Biomedical Research Center, Heinrich-Heine-University Düsseldorf, Universitätsstr. 1, 40225 Düsseldorf, Germany

**Keywords:** Spinal cord injury, Preclinical research, Implants

## Abstract

Traumatic spinal cord injuries result in impairment or even complete loss of motor, sensory and autonomic functions. Recovery after complete spinal cord injury is very limited even in animal models receiving elaborate combinatorial treatments. Recently, we described an implantable microsystem (microconnector) for low-pressure re-adaption of severed spinal stumps in rat. Here we investigate the long-term structural and functional outcome following microconnector implantation after complete spinal cord transection. Re-adaptation of spinal stumps supports formation of a tissue bridge, glial and vascular cell invasion, motor axon regeneration and myelination, resulting in partial recovery of motor-evoked potentials and a thus far unmet improvement of locomotor behaviour. The recovery lasts for at least 5 months. Despite a late partial decline, motor recovery remains significantly superior to controls. Our findings demonstrate that microsystem technology can foster long-lasting functional improvement after complete spinal injury, providing a new and effective tool for combinatorial therapies.

## Abstract (web summary)

Veronica Estrada et al. use implantable microconnectors in rats to promote recovery of complete spinal cord injury. They show that the device leads to re-formation of tissue, vascularization and motor axon regeneration resulting in improvement of locomotor behavior lasting at least 5 months.

## Introduction

After a spinal cord injury (SCI), descending and ascending axons are interrupted resulting in subsequent loss of sensory and motor functions. Severed axons initially attempt to recover via sprouting reactions but spontaneous repair is limited (see ref. ^1^). This is partially explained by inhibitory factors, which accumulate in the glial lesion scar and compromise regenerative responses^2,3^. Many factors contribute to the failure of axonal regeneration within the central nervous system (CNS). These include inflammatory reactions, an unfavourable balance or insufficient amounts of permissive growth substrates and neurotrophic factors, in addition to appearance of numerous inhibitory molecules and catabolic mechanisms in the lesion area that result in very little spontaneous axon outgrowth^4–7^. At the same time, the loss of oligodendrocytes, demyelination of surviving axons and loss of trophic support^8–10^ lead to further damage and impairment of function. The lesion scar, inflammatory reactions and the release of inhibitors can be modulated on a molecular basis, and such possibilities have led to the development of numerous therapeutic approaches to treat SCI^5,9,11–28^. In general, these approaches present only limited possibilities to stabilise and re-adapt injured spinal tissue in submillimetre range.

We have recently described a novel microconnector implant (mechanical microconnector system (mMS)), which was developed for the purpose of re-adapting the separated stumps of severely injured rat spinal cords^29^. The mMS device fosters stabilisation and adaptation of the spinal cord stumps and minimises the distance to be overcome by regenerating axons, while offering the possibility to infuse pharmacological substances directly into the lesion centre via internal microchannels. The mMS connector system is adjustable in size, shape and material.

The complete transection of the spinal cord is a very severe injury model. To date, experimental treatments can, to our knowledge, achieve no or only low degrees of biologically relevant locomotor function recovery following this type of lesion (see, e.g. refs. ^30–36^). Here we show that, after acute severe damage of rat spinal cord by a complete transection at thoracic level T9, mMS implantation results in a significant increase in axonal regeneration across the lesion site, invasion of glial cells and myelination of regenerating axons as well as neovascularisation throughout the implant. Most importantly, in contrast to spinally transected (TX) control rats, the mMS-treated animal group revealed a significantly improved locomotor recovery that persisted for at least 5 months.

The lesion model of complete transection of the spinal cord was chosen for the present proof-of-principle study because it is the only model that provides unequivocal evidence for axonal regeneration. Less severe types of injury (for example, contusion models) generally result in a better functional recovery but at the same time raise the question whether observed improvements are the result of true axonal regeneration or rather compensatory sprouting and functional plasticity. For future preclinical and clinical applications of the mMS device, less severe lesion models will be included.

Here we show the long-term outcome following microconnector implantation after complete spinal cord transection. Re-adaptation of spinal stumps supports tissue restoration, beneficial cell invasion, motor axon regeneration and myelination, leading to a partial recovery of motor-evoked potentials (MEPs) and significantly improved locomotor behaviour.

## Results

### Biocompatibility and integration of the mMS

**Fig. 1:**
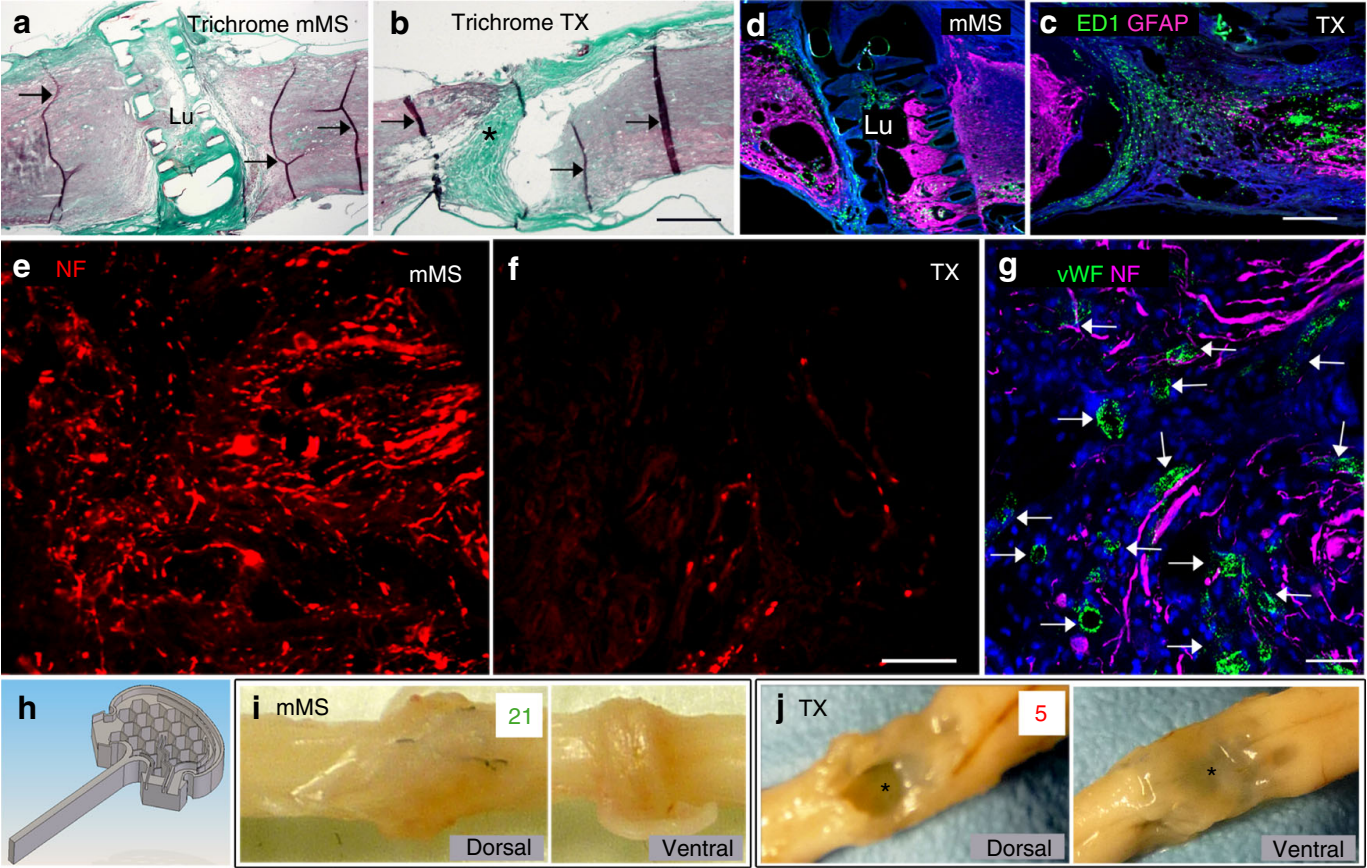
mMS histocompatibility and integration (5 mpo). **a**, **b** Trichrome staining of the lesion area (rostral to the left). Note the loss of tissue and the dense scar (asterisk) in the injury centre of TX animals. The mMS animals exhibit a less condensed scar especially in the mMS lumen (Lu); scarring is evident especially in the outer border regions of the implant; arrows: staining artefacts resulting from tissue creasing during processing. **c**, **d** At the analysed time points, no clear differences in the amount of ED1^+^ macrophages were detected between mMS and TX control animals with immunohistochemical stainings: while in TX controls these cells accumulated particularly in the lesion centre (asterisk), in mMS animals more ED1^+^ cells were detected in the border regions of the mMS compared to the lumen (Lu). **e**, **f** After longer post-implantation time periods, the mMS lumen was filled with numerous NF^+^ axon profiles, whereas the lesion area of TX controls is not attractive for regenerating nerve fibres. **g** Areas rich in vWF^+^ blood vessels (arrows) within the mMS lumen frequently reveal numerous profiles of regrowing axons in close apposition. **h** Schematic representation of one of the complementary halves of the implantable microconnector system. **i**, **j** Representative photographic images of spinal cord specimen of an mMS animal (**i**) and TX control (**j**); cystic cavitation and flattening (asterisk) as well as increased transparency of spinal tissue are recognisable in the lesion area of control animals correlating with decreased mBBB scores (red/green numbers represent the maximum mBBB scores of the respective animals). Scale bars: **a**, **b**: 1 mm, **c**, **d**: 500 µm, **e**, **f**: 100 µm, **g**: 50 µm

To analyse the extent of collagenous lesion scarring after injury, we visualised the connective tissue matrix by trichrome staining (Fig. 1). Compared to the dense collagenous lesion scar of completely TX control animals at 4 months after injury, scarring is much less extensive within the implanted device of mMS animals (Fig. 1a, b). However, the region around the mMS walls still contained some collagenous scar (Fig. 1a). Immunohistochemically, no striking increase in ED1^+^ populations of macrophages or microglia could be observed (Fig. 1c, d). Reactive (glial fibrillary acidic protein (GFAP)^+^) astrocytes were frequently detected in the mMS bridge and lumen regions (Figs. 1d and 4a). Numerous neurofilament (NF)^+^ axon profiles were detected in the mMS lumen (Fig. 1e), whereas much less were found in the lesion centre of TX controls (Fig. 1f; for complete overview of the lesion area of mMS vs. TX animals at lower magnification, see Supplementary Figure 1). The finding of von Willebrand factor (vWF)^+^ endothelial cells revealed the presence of newly formed blood vessels in the lumen of the implanted mMS and showed frequent association of regenerating axon profiles with areas enriched in blood vessels (Fig. 1g). The mMS was well incorporated into the TX spinal cord maintaining its normal diameter (Fig. 1i), whereas spinal cord diameter shrinks in control animals (Fig. 1j).

### Axon regeneration into and beyond the mMS

**Fig. 2:**
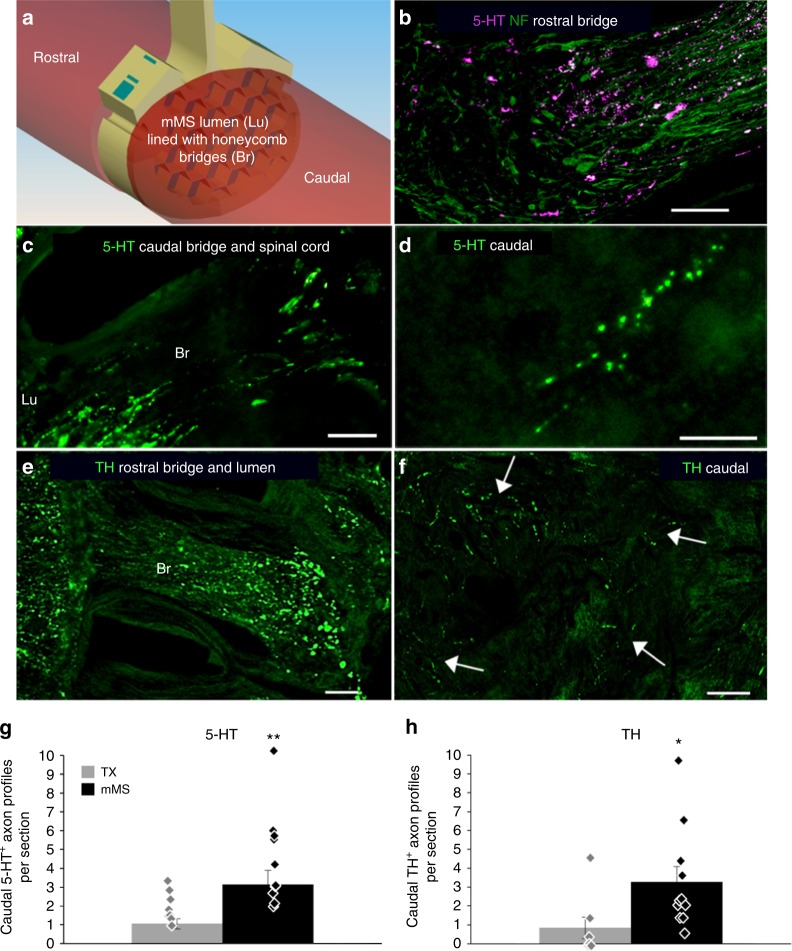
Axon quantification of 5-HT^+^ and TH^+^ profiles. **a** Schematic representation of the implant area with rostral and caudal spinal cord and mMS lumen (Lu) and bridge (Br) regions for better orientation. **b**, **e** Examples of axon ingrowth into the mMS; **c** example of axon outgrowth. **d**, **f** Examples of axon profiles (arrows in **f** indicate TH fibres) that could be detected in the caudal stump of the spinal cord of mMS animals. **g** Quantification of 5-HT^+^ profiles per section in the caudal stump; mean ± SEM; **p = 0.005; Mann–Whitney *U* test. **h** Quantification of TH^+^ profiles per section in the caudal stump, mean ± SEM; *p = 0.023; Mann–Whitney *U* test. Scale bars: **b**, **c**: 100 µm, **d**, **e**: 50 µm, **f**: 20 µm

Since many axonal populations are recognised by general markers for NF, we analysed regenerated axons in more detail by using immunohistochemistry and axonal tracing techniques. Various descending motor axon populations (serotonergic, 5-hydroxytryptamine (5-HT); catecholaminergic, tyrosine hydroxylase (TH)) were investigated immunohistochemically. In addition, intra-spinal rostral biotinylated dextran amine (BDA) tracing, and anterograde corticospinal tract (CST) BDA tracing were used to unambiguously detect regenerated descending axons. 5-HT and TH stainings were chosen as examples for axonal populations that are involved in locomotor function. 5-HT^+^ and TH^+^ axon profiles were detected both in the mMS lumen and in the adjacent caudal spinal cord (Fig. 2a–f). Quantification revealed that substantially more axons had regenerated across the mMS-implanted lesion as compared to the TX injury alone (Fig. 2g, h; Supplementary Figure 2). Despite the possibility that some of the detected 5-HT- and TH-positive axons might be derived from serotonergic or catecholaminergic propriospinal interneurons^37–39^, the observed differences show that mMS implantation led to a detectable three-fold increase in these motor axon profiles in the caudal spinal cord.

**Fig. 3:**
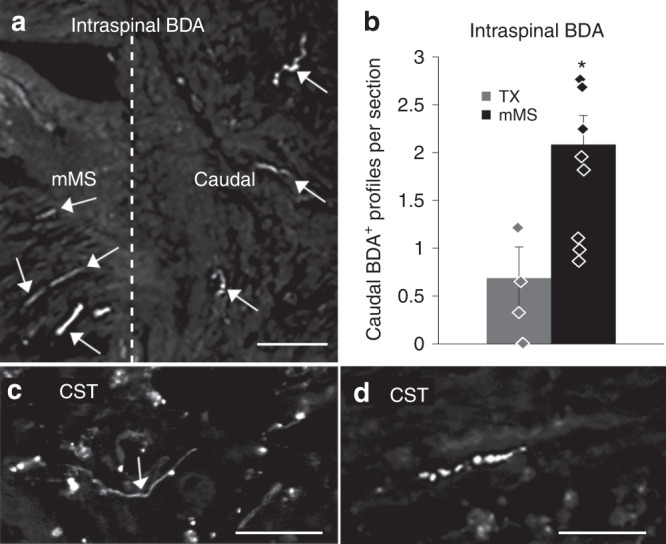
Anterograde BDA labelling of regenerating axons. **a** Example of rostral intraspinal BDA-labelled axons, which have regenerated from the mMS into the caudal spinal cord. The dashed line indicates the mMS/spinal cord interface. **b** Quantification of axon profiles per section in the caudal stump that have been traced via rostral intraspinal BDA labelling; mean ± SEM. **c**, **d** Examples of BDA-labelled CST axon profiles at 0.5 mm (**c**) and ca. 3 mm caudal from the mMS implant (**d**). Scale bars: **a**: 50 µm, **c**, **d**: 25 µm; Mann–Whitney *U* test: *p = 0.042

Intraspinal rostral BDA labelling to validate fibre regeneration proved that re-adaptation of the spinal stumps by the mMS supports axon growth not only into but also across the lesion site. The tracer is taken up by axons, which pass through the site of the BDA injection rostral to the lesion. We found that significantly more BDA-labelled axons were able to grow into the caudal spinal cord of mMS animals compared to TX controls (Fig. 3a, b). Following cortical BDA anterograde tracing in three mMS animals, we found that even CST axons, which are known for their poor regenerative capacity, grew across the mMS bridge into caudal spinal cord areas (Fig. 3c, d).

### Schwann cell association and myelination of regrown axons in the mMS

**Fig. 4:**
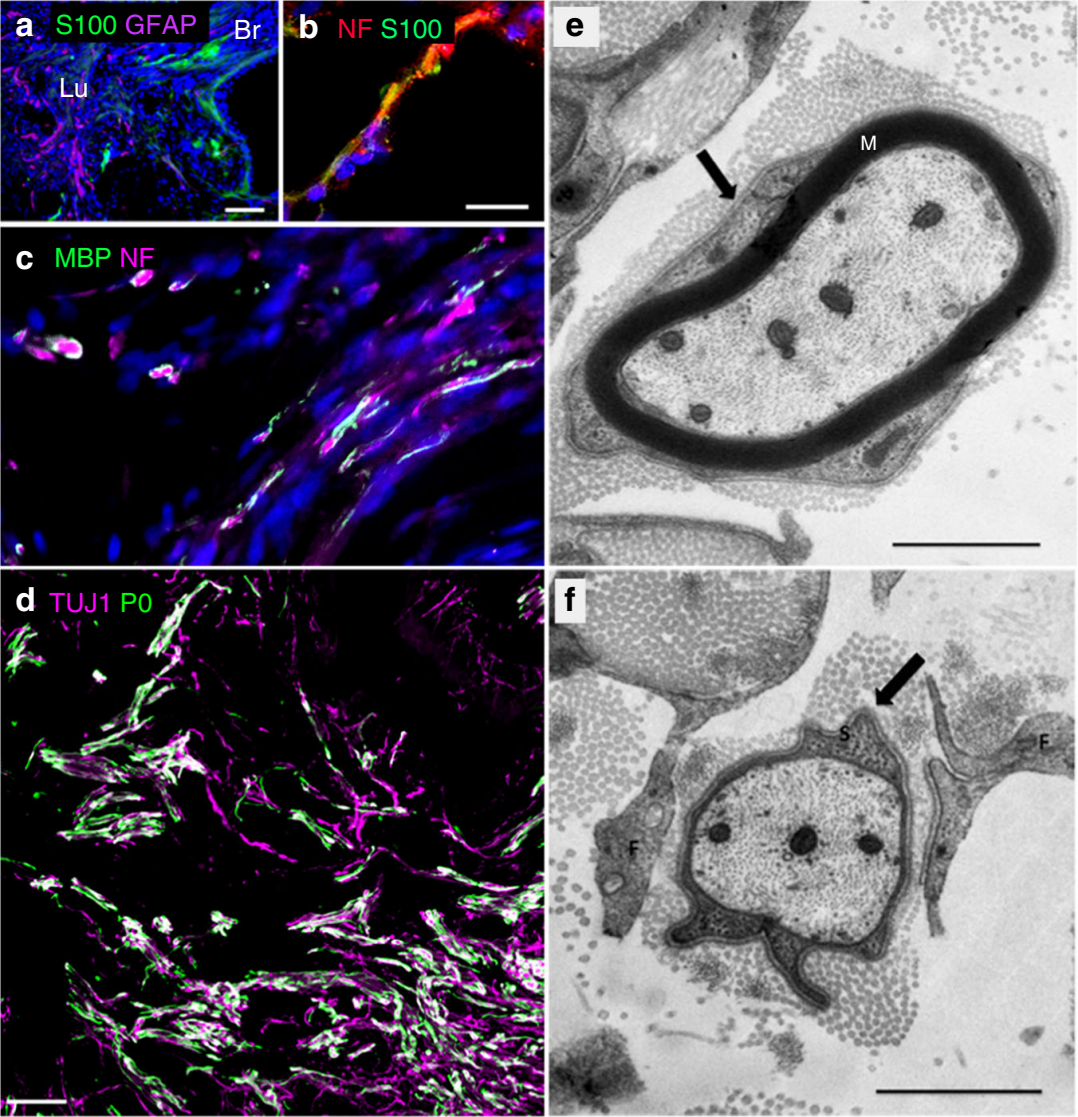
Association of Schwann cells with regenerated axons in the mMS lumen. **a** Both Schwann cells (S100^+^/GFAP^−^) and astrocytes (S100^−^/GFAP^+^ or S100^+^/GFAP^+^, respectively) invade the mMS lumen. **b** Close association of NF^+^ axons with S100^+^ Schwann cells. **c** Co-staining of NF and MBP indicates myelination of axons in the mMS lumen. **d** Numerous axons (TUJ1^+^) in the mMS lumen are myelinated with peripheral myelin (P0^+^). **e** Electron micrograph of a myelinated regrown axon in the lumen of a mMS honeycomb. Note basal lamina (arrow), identifying the myelinating cell as Schwann cell-like glial cell. M myelin. **f** Electron micrograph of an axon in the lumen of an mMS honeycomb, ensheathed by a pro-myelinating glial cell. A basal lamina (arrow), identifies the ensheathing cell as a Schwann cell-like glial cell. M myelin. Scale bars: **a**: 100 µm; **b**, **c**: 20 µm; **d**: 50 µm; **e**, **f**: 1 µm

Numerous S100^+^/GFAP^−^ Schwann cells were detected in the mMS lumen (Fig. 4a) and often (but not always) appeared in close proximity to axons (Fig. 4b, Suppl. Figure 3). Interestingly, at 5 months many axon profiles in the mMS lumen appeared to be myelinated as indicated by myelin basic protein (MBP) immunoreactivity (Fig. 4c). Positive staining for myelin protein zero (P0), a specific marker of Schwann cell myelin, further indicates that most, if not all, of the detected myelin in the mMS belonged to Schwann cell-like cells (Fig. 4d). Axonal ensheathment by compact myelin derived from basement membrane-bearing Schwann cell-like cells as well as wrapping of single axons by pro-myelinating Schwann cell-like cells was confirmed by transmission electron microscopy (Fig. 4e, f). Co-localisation of P0 immunoreactivity with TH^+^ or 5-HT^+^ neurons was not detected in the analysed areas, suggesting that the myelinated axons presumably emanate from other fibre populations.

### Improved locomotion after mMS implantation

Functional locomotor outcome was tested in the open field once per week over a period of at least 5 months. During the entire observation period, animals of the mMS-implanted group performed better with respect to locomotor outcome than TX control animals. In the regular Basso–Beattie–Bresnahan (BBB)^40^ open field test, mMS animals reached scores of 8–10.5, relating to sweeping movements of both hindlimbs or to occasional weight-supported stepping without forelimb–hindlimb coordination, whereas the control group reached BBB scores of 2–4 only. However, the regular BBB open field test—which was originally developed for moderate thoracic contusion injuries—does not allow any further discrimination and evaluation of subtle improvements that occur after TX. Therefore, we used a recently modified BBB (mBBB) score, which is better suited for rats with a complete transection of their thoracic spinal cord^41^.

**Fig. 5:**
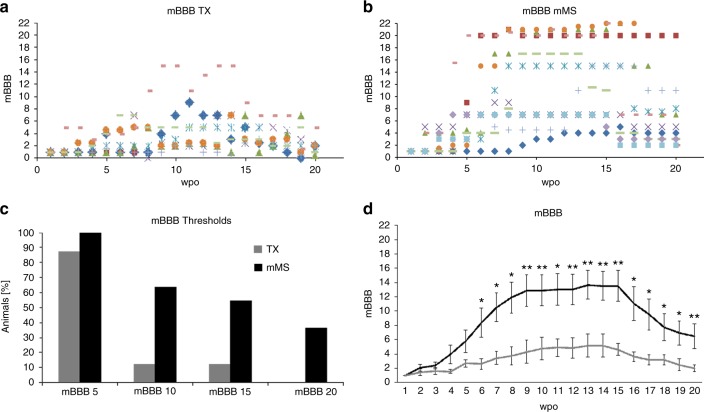
Functional locomotor improvement after acute mMS implantation. **a**, **b** Scatter diagrams of the individual mBBB scores of the TX control rats and mMS-treated animals. Each symbol represents an individual animal. Owing to the loss of animals (see online methods for details), the depicted diagrams are based on the following numbers of animals per respective time point: TX, wpo 1–7: 9 rats, wpo 8–20: 8 rats; mMS: wpo 1 and 2: 12 rats, wpo 3–17: 11 rats, wpo 18–20: 10 rats. **c** mBBB threshold analysis; **d** Average mBBB scores over the 5 months (20 weeks) period, mean ± SEM (see individual data points in **a** or **b**, respectively). Note the drop in the mBBB scores at later time points. Mann–Whitney *U* test: *p < 0.05, **p < 0.01 (*p* values: wpo 6: 0.02, wpo 7: 0.01; wpo 8: 0.01; wpo 9: 0.007; wpo 10: 0.007; wpo 11: 0.01; wpo 12: 0.009; wpo 13: 0.007; wpo 14: 0.008; wpo 15: 0.005; wpo 16: 0.03; wpo 17: 0.02; wpo 18: 0.04; wpo 19: 0.02; wpo 20: 0.007)

**Fig. 6:**
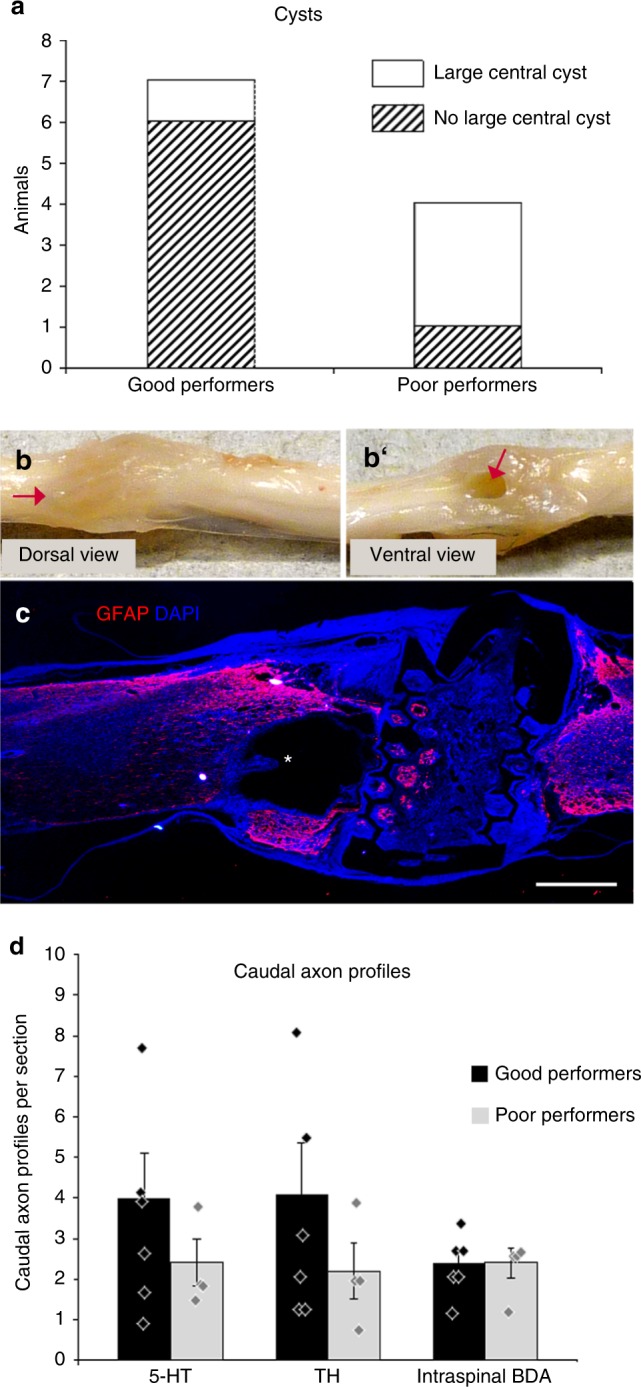
Occurrence of large cysts and caudal axon profiles in good vs. poor performers of the mMS group. **a** Only 1 out of 7 good performers (14%) compared to 3 out of 4 poor performers (75%) revealed a large central cyst. **b**, **b**', **c** Large cyst formation (indicated by red arrows in **b**, **b**' and asterisk in **c**) in the spinal cord of a poor performer (mBBB 9) that showed high numbers of caudal 5-HT^+^ and TH^+^ axon profiles; the cystic cavity was already apparent prior to tissue processing (photographic images in **b**, **b**'). **d** Quantification of axon profiles suggests a generally increased (doubled) number of both 5-HT^+^ and TH^+^ profiles in the caudal spinal cord of the good performers in comparison to poor performers, while no difference between good and poor performers was detected regarding the numbers of intra-spinally BDA-labelled axon profiles. Scale bar in **c**: 1 mm

Animals of the mMS group reached considerably higher mBBB scores than controls (Fig. 5). This is revealed both by the scatter diagrams (Fig. 5a, b) showing the performance on the level of individual animals and by mBBB threshold analyses (Fig. 5c). All mMS animals reached the lowest threshold of mBBB 5, and notably higher percentages of mMS compared to TX animals reached the threshold of mBBB 10 (66 vs. 12.5%), respectively. While six rats (50%) of the mMS-treated group achieved a maximum mBBB score of ≥15, only one control animal (12.5%) reached an mBBB score of 15. Four animals (33%) of the mMS group, but none of the controls, were able to reach the highest threshold of mBBB 20 (Fig. 5a–c). The latter four mMS animals were by far the best performers and, indeed, were the first reaching or passing a locomotor score of mBBB 15 within 4–7 weeks after treatment (Fig. 5b). Interestingly, we also noticed a correlation between locomotor improvement and preservation of the spinal cord tissue and diameter as provided by the mMS (examples in Fig. 1i, j; also see Fig. 6).

The average performance of the two animal groups throughout the entire open field study is summarised in Fig. 5d. At 4 weeks after mMS implantation, the animals achieved mean mBBB scores of 4 (“occasional right–left alternation of the hindlimbs with weak amplitude”). After 7 weeks, mMS animals reached mean mBBB scores of ≥10, indicating that the movements of their hindlimbs included occasional, frequent or consistent weight support. Subsequently, the animals scored increasing mBBB values and plateaued at mean mBBB 13–14 (“occasional to frequent right–left alternation of the hindlimbs with large amplitudes and either some body weight (bw) support or some plantar foot placement”) lasting for several weeks (weeks post-opearation (wpo) 9–15). From week 6 onward until to the end of the study, the mMS group performed consistently and significantly better than the TX control group. Throughout the entire observation period the average score of the TX control group stayed below mBBB 6 (“frequent alternation of the hindlimbs with weak amplitude”). Although the locomotor scores of animals in both the mMS and control groups started to decline in wpo 16, the mMS animals continued to perform significantly better than TX control animals until the end of the study (Fig. 5d).

Upon locomotor performance, the animals of the mMS-treated group could be subdivided into “good performers” (7 rats; mBBB ≥15) and “poor performers” (4 rats; mBBB ≤10). Closer inspection of the two subgroups revealed occurrence of large cysts in three of the four poor performers compared to only one of the seven good performers (Fig. 6a). Correspondingly, the numbers of 5-HT^+^ and TH^+^ axon profiles in the caudal spinal cord of the good performers was detected to be twice as high as those of the poor performers (Fig. 6d). Surprisingly, one poor performer, which exhibited high numbers of axon profiles in the caudal spinal cord only, revealed a maximum mBBB score of 9. This incongruity could be attributed to a large cyst, which was detected adjacent to the rostral border of the mMS implantation site (Fig. 6b, b’, c).

**Fig. 7:**
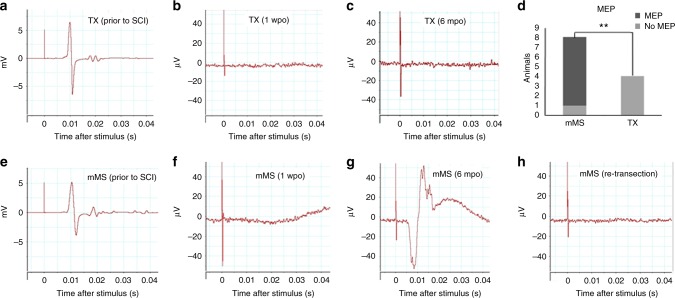
Representative MEP measurements from mMS-treated vs. control animals. **a** Strong MEP signals could be recorded from TX animals prior to SCI. **b**, **c** No signals were recorded from TX-animals at early (**b**, 1 wpo) and late (**c**, 2–6 mpo) post-injury time points, respectively. **d** MEPs could be recorded from 7 (out of 8) mMS animals but in none of the 4 TX animals. **h** Summary of MEP recordings. In 7 out of the 8 mMS animals, MEP signals could be recorded, whereas in 0 out of the 4 TX control animals MEP signals were detected; Fisher Exact Test, **p = 0.01; **e** Prior to SCI and treatment, clear MEP signals could be recorded from mMS animals. **f**, **g** While no signals could be recorded at early post-treatment time points (**f**, 1 wpo), clear albeit weak (18–105 µV) MEP signals were detected after longer post-treatment times in mMS-treated animals (**g**, 2–6 mpo). **h** The regained MEP signal in mMS-treated rats disappeared after complete re-transection of the spinal cord at T10

Locomotor improvement observed in mMS animals compared to control rats could be underscored by electrophysiological recordings (Fig. 7) in a separate experiment. In TX control animals (*N* = 4), which also revealed strong MEP signals prior to the injury (Fig. 7a), no MEP signals could be detected at early (1 wpo; Fig. 7b) or late post-injury time points (2–6 months post-operation (mpo); Fig. 7c). Similarly, mMS-treated animals (*N* = 8) also revealed clear MEP signals prior to the injury (Fig. 7e) and no MEP conduction at early post-treatment time points (1 wpo; Fig. 7f), indicating the completeness of spinal transection. However, at late post-treatment time points (2–6 mpo), weak but clear MEP signals (18–105 µV) with a threshold of 15 µV could be recorded from 7 mMS animals (Fig. 7g, d). Re-gained MEP signals disappeared after re-transection of the spinal cord (Fig. 7h).

## Discussion

Following complete spinal transection, the functional locomotor outcome of severely injured rats could be improved by low-pressure mechanical re-adaptation and stabilisation of the stumps using a biocompatible microconnector device, mMS, which integrated well into the spinal cord, supported formation of a vascularised tissue bridge and prevented a reduction of spinal cord diameter. The mMS fostered tissue preservation and stabilisation of the spinal cord around the lesion area, which corresponded to high mBBB scores (see example in Fig. 1i). TX controls frequently exhibited spinal cord lesion areas that contained either cystic cavities or spinal cord tissue in the respective area obviously vanished, likely due to secondary degeneration, as recognised by the flattened and transparent appearance of spinal cord specimens (see example in Fig. 1j). No cases of reduction in spinal cord diameter were observed in mMS-treated animals. Beneficial effects of tissue protection on functional outcome have been demonstrated previously^42,43^.

Interestingly, bringing the severed spinal stumps in close apposition merely by mechanical forces using the mMS was sufficient to promote regeneration of severed axons *into* and also *beyond* the lesion area. Regenerated axons were detected by NF immunostaining within the mMS and by intraspinal anterograde BDA tracing of descending axons as well as by anterograde cortical tracing of CST axons. Beyond the lesion, traced axons were identified in intact caudal spinal cord regions. Further immunohistological characterisation revealed that both serotonergic and catecholaminergic axon populations were among the regenerated fibres. Our data indicate that, compared to the low proportion of spontaneously regenerating serotonergic and catecholaminergic axons in TX control animals, implantation of the mMS resulted in significantly increased numbers (three-fold increase) of the respective axon profiles in the caudal spinal cord. The latter axon profiles could arise from the raphe nucleus or the locus coeruleus, respectively, or originate from propriospinal interneurons^37–39^. The detection of traced CST axons growing beyond the mMS into the caudal stump was intriguing because corticospinal axons are known for their poor regenerative capacity.

Regenerated axons were frequently associated with blood vessel-rich areas in the mMS lumen, suggesting a beneficial effect of angiogenesis on axon growth. It is known that blood vessels are necessary for tissue regeneration and for supply and exchange of nutrients as well as chemical signalling and thus considered vital for repair and survival of regenerating nerve fibres^44–46^. Moreover, blood vessels have even been shown to serve as tracks to direct migrating Schwann cells in regenerating peripheral nerve^47^. This view is in line with our observation that regenerating axons within the lumina of mMS devices were in close association with Schwann cells, which are known to foster axon regrowth^48,49^, and most likely myelinate the regrown axons, later, as revealed by immunohistochemistry and transmission electron microscopy. Abundant P0 immunoreactivity in combination with basement membranes surrounding the myelinating or pro-myelinating glial profiles unequivocally identify a Schwann cell-like phenotype of still uncertain origin. It is possible that the dural rupture caused by the transection injury may allow peripheral Schwann cells—presumably originating from adjacent nerve roots—to invade the lesion area^50,51^. Alternatively, the detected Schwann cells might be derived from Schwann cell precursors^52,53^, originate from bone marrow^54^, or emanate from endogenous CNS-resident glial progenitors^55^.

Despite the fact that the original BBB open field analysis^40^ is not well suited to animals with a total transection of the spinal cord^41,56^, it is still applied in numerous animal studies that employ complete spinal lesions. Numerous rodent studies published in the past 15 years using original BBB scores reported recovery rates after acute and sub-acute complete thoracic spinal cord transection and various (single as well as combinatorial) therapeutic treatments in the range of BBB 3–8^30–36^, respectively, all staying well below the plateau of locomotor recovery, reached by implantation of the mMS (mBBB of 13–14, corresponding to a BBB score of 10–10.5).

Our data demonstrate that mMS-implanted animals were able to reach a hitherto unattained level of recovery in hindlimb locomotor function following complete spinal cord transection. Animals of the mMS-implanted group performed robustly and (from wpo 6 onwards) significantly better in the open field than control animals. The improved functional outcome of the mMS group corresponds to the presence of increased numbers of regenerating axons in the caudal spinal cord of treated animals. Within 4 weeks after mMS implantation, the animals achieved mBBB scores, which relate to and confirm the BBB scores that were detected in our initial short-term mMS study^29^. After 7 weeks, mMS animals reached even higher mBBB scores, indicating that their hindlimb movements involved occasional, frequent or consistent weight support. The animals continuously scored increasing values for the following weeks and plateaued at performance levels, which included occasional to frequent right–left alternation of the hindlimbs with large amplitudes and either some bw support or some plantar foot placement over several weeks (wpo 9–15).

Very interestingly, upon closer inspection the mMS-treated group appears to be split into extremely well-performing animals (good performers) and those showing a course of recovery largely resembling control animals (poor performers). A remarkable proportion of the mMS animals (4 rats, 36%) even reached the highest mBBB scores of mBBB 20–22 corresponding to a classical BBB score of 10–11. TX control animals never reached average mBBB scores higher than mBBB 6. As the only outlier, one control animal showed a spontaneous but transient locomotor score in the range of mBBB 10–15 between wpo 8 and 14. The overall weak locomotor functional outcome observed in control animals compared to the overall strong improvement found in the mMS animals could possibly be explained by the lower numbers of regenerating serotonergic and/or catecholaminergic fibres regenerating in controls.

Further subdivision of the mMS-treated animals revealed the negative influence of large cysts in or near the lesion area on locomotor functional outcome: The good performers did—with one exception—not exhibit the formation of a large cyst in the lesion area and their locomotor functional outcome could be attributed to the enhanced amounts of the detected regenerating axon profiles in the caudal spinal cord. The majority of the poor performers, however, exhibited a large cyst adjacent to the mMS implant, which might impede regenerative axon growth and decrease the mechanical stability of the tissue. Similar large cysts were commonly found in the lesion area of the spinal cord of TX control animals. One animal of the mMS subgroup of poor performers revealed surprisingly high numbers of both 5-HT^+^ and TH^+^ axon profiles despite formation of a large cyst. Presumably due to the large cyst this animal could not perform as well in the mBBB locomotor test as animals of the good performer group, which lack cyst formation. Since the number of intraspinally BDA-labelled descending axon profiles in the caudal spinal cord of both the good- and poor-performing mMS-treated subgroups did not differ, these axons, in contrast to the 5-HT^+^ and TH^+^ subpopulations of fibres, are less likely to contribute to the observed differences in locomotor outcome.

MEP recordings confirmed the long-term functional improvement following mMS treatment. The fact that only mMS animals, in contrast to TX controls, were able to recover their MEP signal conduction to some, albeit low, degree during the long-term recovery period further underscores the suitability of the microconnector treatment to improve functional outcome. The finding that the MEP signals disappeared after re-transection of the spinal cord is in line with a potential functional role of axonal reconnectivity for the achieved locomotor improvement after mMS treatment.

The late decline in locomotor scores starting at wpo 16 in both the mMS treated as well as control group is not yet fully understood. Muscle atrophy^57–59^ is likely to cause the decrease in locomotor performance since leg muscle use is initially completely lacking after SCI and still remains rather low thereafter. In a different study, we compared weight and girth of the *tibialis anterior* muscle of SCI animals at 8 months after a complete spinal cord transection and uninjured littermates. The comparison of the average girth and weight revealed considerable—nearly 50%—muscle atrophy following spinal transection [TX animals (*n* = 18): 4.3 cm girth, 13.5 g weight; uninjured controls (*n* = 3): 7.8 cm girth, 23 g weight]. On the other hand, maladaptive plasticity^60^ or degradation of transient synaptic contacts due to axonal pruning could also be responsible for the observed decline in functional outcome.

It has been reported that rehabilitative measures like treadmill training and enriched environment potentially preserve and support residual or regained locomotor function after experimental SCI^60–62^. Hence the late decline in locomotor outcome we observed in our long-term behavioural study could possibly be prevented in future by applying concomitant treadmill training in addition to implantation of the microconnector. A comparable decline in locomotor function after an initial recovery phase following treatment has been described previously^63^. Moon et al. attributed the observed loss of initial locomotor recovery after a prolonged time period of approximately 3 months post-SCI and treatment to a lack of hindlimb usage and a general decrease in locomotion, which could be overcome by the provision of motor enrichment housing instead of standard housing^63^.

Owing to its internal microchannel system for controlled local drug delivery and cell infusion, the mMS microsystem is well suited for a wide range of combinatorial therapies comprising antibodies against repulsive molecules, iron chelators, trophic factors, enzymes and other compounds^64–77^. The currently limited functional outcomes of preclinical molecular and cellular therapies applied to severe/complete SCI could probably be substantially improved in combination with the mMS microconnector device serving as a basic treatment. Future biodegradability and individual adaptation of the mMS to match shape and size of individual spinal lesions as determined by imaging techniques like magnetic resonance imaging scans, and fabrication of the device via three-dimensional printing technology offer possibilities for both standardised and personalised medical treatment of acute and chronic severe SCI patients.

## Methods

### mMS design

Design and fabrication of the implantable microconnector systems (mMS) have recently been reported in detail^29^. Microconnectors used in this study were fabricated with polymethyl methacrylate. Connector systems with small holes (cf. ref. ^29^; see schematic representation in Fig. 1a) were used for the study described here.

### Experimental animals and surgical procedures

For this study, adult female Wistar rats (HanTac:WH; Taconic and RjHan:W) weighing 230–250 g were used. Animals were housed in groups of four animals per cage with food and water ad libitum. Although over the course of the study all animals revealed slightly varying degrees of weight gain, no difference was observed between mMS-treated and TX control animals in this respect.

### SCI and mMS implantation

Institutional guidelines for animal safety and comfort were adhered to, and all surgical interventions including electrophysiological recordings and pre- and post-surgical animal care were provided in compliance with the German Animal Protection law (AZ 8.87–50.10.34.08.061, AZ 84–02.04.2011.A332, AZ 87–51.04.2011.A023, AZ 84–02.04.2014.A195, State Office, Environmental and Consumer Protection of North Rhine-Westphalia, LANUV NRW).

A complete thoracic spinal cord transection was performed at thoracic level T9. Compared to partial injuries, this lesion model holds several advantages: it is highly reproducible and leads to comparable degrees of functional loss in all animals. Therefore, locomotor improvements can be detected and followed up very accurately. Details of the performed surgical procedures have been described recently^78^. In summary, following laminectomy of the vertebrae Th8 and Th9 and subsequent complete transection of the thoracic spinal cord by a transverse cut using fine eye scissors, the mMS was fixed and implanted into the tissue gap between the severed stumps, as described^78^. Two small spatulas were used to carefully pull the segments apart to ensure completeness of transection. After suturing the dura mater above the mMS implant, the adjacent spinal cord stumps were sucked into the lumen of the device via gentle negative pressure (200–250 mbar, 10 min). Finally, overlying connective and muscle tissues were sutured in layers.

Control animals (TX) received a complete transection of the spinal cord and the subsequent dura suture but no mMS implant. Both animal groups, which were included in the locomotor functional study (TX vs. mMS), initially consisted of *N* = 12 rats each. One TX animal was sacrificed in wpo 7 (TX) because it had developed a bladder infection, which remained unresponsive to antibiotic treatment. Three animals of the TX group died unexpectedly in the first and one mMS animal in the second post-operational week. These animals most likely died from post-surgical complications after the severe injury. One animal of the mMS group died unexpectedly late in wpo 18 for unknown reasons.

### Recording of MEPs

Under ketamine/xylazine anaesthesia, screws were implanted into the skull of the rats, based on a protocol by Schlag et al.^79^. Briefly, after opening the skin two holes (diameter ca. 1 mm) were drilled into the scull and screws (outer diameter of shank 1.2 mm) were screwed into these holes. One screw (positive pole) was implanted into the left hemisphere above the hindlimb region of the sensorimotor cortex (2.4 or 2 mm lateral from the midline, Bregma −2.0 or −2.5 mm; 0.75–1 mm depth). A second screw (negative pole) was implanted above the cerebellum (0 mm lateral from the midline, Lambda −1.5 mm, 1–1.5 mm depth). For MEP recordings, the rats were anaesthetised with ketamine (55 mg/kg bw)/xylazine (5 mg/kg bw), intraperitoneally. The skin above the screws was re-opened and custom-made electrode wires were coiled around the exposed screws. For recording of MEPs, needle electrodes (MLA1204, AD instruments) were inserted into the belly of the musculus tibialis anterior with a reference placed subcutaneously (s.c.) into the sole of the paw. The grounding electrode was inserted s.c. at the animal’s back (at the height of the hips). MEPs were recorded under light anaesthesia (according to Zandieh et al.^80^). Stimulation and recording were performed with a PowerLab26T (AD Instruments). Stimuli (duration 100 ms) were increased in 1 mA steps starting at 1 mA until the resulting MEP showed no further increase. Final MEP recordings were performed 1 or 2 days prior to sacrifice. Since movements of the animal’s body could lead to false positive results (due to movement of the needle electrodes), care was taken not to include corresponding signals in the analysis.

### Behavioural testing

Locomotor behaviour of animals was assessed weekly using a modified version of the BBB open field score (mBBB) for complete spinal cord transection^41^. To evaluate the locomotor outcome, each animal was observed in an open field for 4 min by two observers, and its movements were rated accordingly. Behavioural testing was carried out blinded to the treatment of the animals and was performed over a time period of 5 months after the initial surgery.

### Rostral intraspinal labelling of descending axons in the thoracic spinal cord

Rostral intraspinal tracing with BDA (10,000 MW, 10%; Life Technologies) was performed 1 week prior to sacrifice as described previously^41^. BDA was injected into the spinal cord 3 mm rostrally to the lesion/implantation site of anaesthetised animals. Injections (0.2 μl each) of BDA were applied at the following spinal coordinates: In the midline at a depth of 1.5 mm (ventral funiculus), 0.8 mm (dorsal CST) and 0.5 mm (cuneate and gracile funiculi) from the pia mater, and at 1.0 mm laterally from the midline on each side at depths of 1.2, 0.8 and 0.4 mm (lateral funiculi) from the pia mater using a grease-sealed glass cannula attached to a Hamilton syringe.

### Anterograde CST tracing

Anterograde CST tracing was performed 3 weeks prior to sacrifice. After craniotomy, the CST of anaesthetised animals was anterogradely traced via eight small volume injections (0.2 μl each) of BDA into the sensorimotor cortex of each hemisphere using the Kopf stereotactic frame and coordinates previously described^41,70^.

### Re-transection of the spinal cord

Three days prior to sacrifice, a complete transection was performed adjacent to the caudal border of the lesion/mMS area of 3 mMS animals. Subsequently, the dura was sutured.

### Tissue preparation

Tissue preparation for immunohistochemistry was performed as previously described:^41,70^ The animals were anaesthetised with Narcoren® (Merial) and transcardially perfused with ice-cold phosphate-buffered saline (PBS) for 2 min and subsequently with 4% paraformaldehyde (PFA; Merck) for 15 min. The spinal cords were post-fixed in 4% PFA at 4 °C. Spinal cord specimens containing the lesion area were embedded and cut in paraffin (20 μm parasagittal sections).

The extent of the SCI scar tissue was analysed via Masson’s trichrome histological staining.

### Immunohistochemistry

Immunohistochemical staining of paraffin sections was preceded by deparaffinisation, and standard immunohistological protocols were used for all staining procedures. Untreated sections and control sections lacking primary antibody were analysed for evidence of auto-fluorescence. All sections for immunohistochemical analyses were taken from animals with survival times of 5–7 months.

The following antibodies directed to the proteins mentioned were used: SMI312 (pan-axonal marker of phosphorylated NF; Covance, 1:1000), Neuronal Class III β-Tubulin (TUJ1; Covance, 1:1500), glial fibrillary acidic protein (GFAP; Millipore, 1:1000), glial fibrillary acidic protein (GFAP; Dako, 1:1000), ED1 (Serotec, 1:1000), MBP (Millipore, 1:50), P0 (Aves Labs, 1:100), 5-HT (Biologo, 1:30), TH (Abcam, 1:750), vWF (Dako, 1:1000), and S100β (S100; Sigma, 1:1000). Epitope retrieval with citrate buffer was used for MBP, and epitope retrieval with protease was used for vWF. Sections were stained with one of the antibodies listed above by standard immunohistological protocols: Washing with PBS, blocking with 5% normal donkey serum for 1 h at room temperature, incubation with first antibody in PBS/5% normal serum overnight at 4 °C, washing with PBS, and incubation for 1 h with Alexa 488- or 594-conjugated secondary antibody (Molecular Probes), respectively. For BDA visualisation of anterogradely labelled axons, Oregon Green® 488 (Molecular Probes; 1:1000) was applied.

For histological trichrome staining, following deparaffinisation and rehydration, sections were immersed in plasma stain solution A (containing acid fuchsin, Xylidine Ponceau and acetic acid), solution B (phosphomolybdic acid solution) and fibre stain solution C (containing Light Green SF yellowish and acetic acid) for 10 min each.

The sections were analysed and/or photographed with either a Keyence Biozero 8000 microscope or a Nikon Diaphot 300 fluorescent microscope or a Zeiss LSM 510 Meta confocal microscope.

### Quantification of axon profiles

The quantification of axon profiles was performed blinded to the treatment of the animals using sections from animals with a survival time of 5–7 mpo. For quantification of 5-HT- and TH-positive axon profiles in the spinal cord caudal to the lesion (an area of approximately 1 cm in length starting from the lesion border was evaluated), on average 10 TH and 13 5-HT immunostained 20 μm parasagittal sections from analogous regions were taken per animal. On average, six sections from analogous regions of each of these respective animals were used for the quantification of axon profiles that had been labelled via rostral BDA injections. Sections were analysed using the ×20 objective of a Nikon Diaphot 300 fluorescent microscope. As described in ref. ^41^, whenever the identification of single axon profiles could not be ensured (e.g. in small entangled axon bundles) the ×40 objective was used for confirmation. Axon profiles were counted under the microscope.

### Transmission electron microscopy

For electron microscopic analysis, mMS of operated rats were postfixed in perfusion fixative and cleaned from surrounding connective tissues, followed by osmification and embedding in Epon^41^. Semithin sections were prepared and inspected by light microscope to select internal structures of the mMS containing nerve fibres. For ultrathin sections, mMS material was trimmed away with a razor blade, sparing the lumen of the most innervated honeycomb. Ultrathin sections were investigated by a ProScan Slow Scan CCD (ProScan) camera mounted to a Leo 906 E electron microscope (Zeiss) and the corresponding software iTEM (Soft Imaging System).

### Statistics

StatPlus Software was used for the majority of the statistical intergroup comparisons. For the MEP study, SigmaStat was used for the statistical analyses. As reported in ref. ^41^, data sets were tested for significant deviations from statistical normality using Shapiro–Wilk test (*p* = 0.05). Because this test revealed that the distributions of the data were not normal, nonparametric Kruskal–Wallis test was used for evaluation of significant intergroup differences in axon numbers. Nonparametric tests are better suited for statistical analyses of data that contain outliers. The experimental groups were considered significantly different at *p* ≤ 0.05. For the statistical analysis of the behavioural test results of animals with complete spinal transection, a paired comparison was performed using the one-sided Mann–Whitney *U* test. The experimental groups were considered significantly different at *p* ≤ 0.05. For the electrophysiological analysis, the statistical method used was Fisher Exact Test.

## Electronic supplementary material

Supplementary Figures

## Data Availability

The authors declare that the data supporting the findings of this study are available within the paper. All source data are available on request (locomotor behaviour: V.E.; mMS: H.K.T.; MEP recordings: J.K.; electron microscopy: R.M.; general: H.W.M.).
