# Supplementary material for: Low-pressure micro-mechanical re-adaptation device sustainably and effectively improves locomotor recovery from complete spinal cord injury
Source: Commun Biol. 2018 Nov 26;1:205. doi: 10.1038/s42003-018-0210-8 (PMC6255786; doi:10.1038/s42003-018-0210-8)
Supplement: Supplementary file 1 — Supplementary Figures [file 42003_2018_210_MOESM1_ESM.pdf]

Supplementary Figures

Supplementary Figure 1:

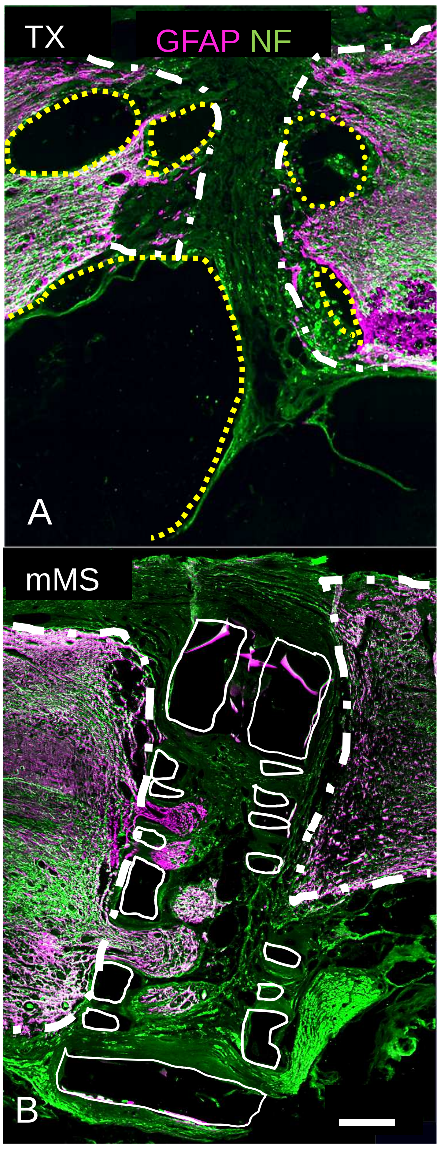

Legend Supplementary Figure 1: Overview of NF staining in the lesion area at 6 mpo. A: TX control; B: mMS. Bold white dashed lines mark the borders of the spinal cord tissue adjacent to the lesion/implantation area. Cystic cavities are marked by yellow dashed lines in A. A large cystic intradural cavity has displaced large amounts of the rostral spinal cord tissue in a TX animal. Small white dashed lines in B mark tissue-free areas in the mMS region, which are caused by loss of the PMMA during tissue processing. PMMA residues can be recognised by their strong unspecific fluorescence in the large top and bottom holes. Scale bar: 250 µm.

Supplementary Figure 2:

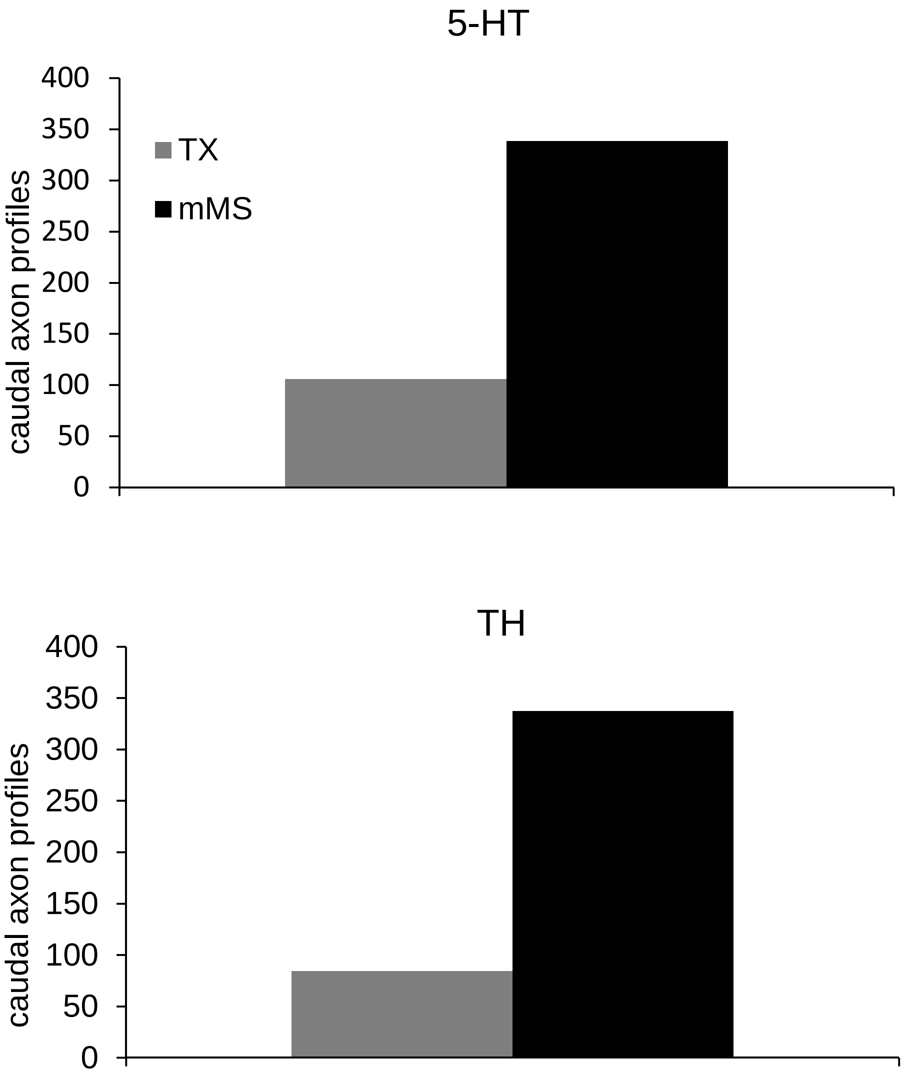

Legend Supplementary Figure 2: Extrapolation of caudal axon profiles. A calculation was performed based on the axon numbers counted in the exemplary series of 20 µm thick sections (on average 10 sections for TH fibres and 13 sections for 5-HT fibres per animal, respectively) spanning the entire width of the spinal cord. Axon numbers were then extrapolated for the populations of 5-HT and TH fibres for the entire width (approx. 2 mm) of the thoracic spinal cord of adult female Wistar rats for a hypothetical number (n = one hundred) 20 µm sections. Since this is an extrapolation of not normally distributed data shown in Fig. 2g and 2h we have omitted statistical analysis.

Supplementary Figure 3:

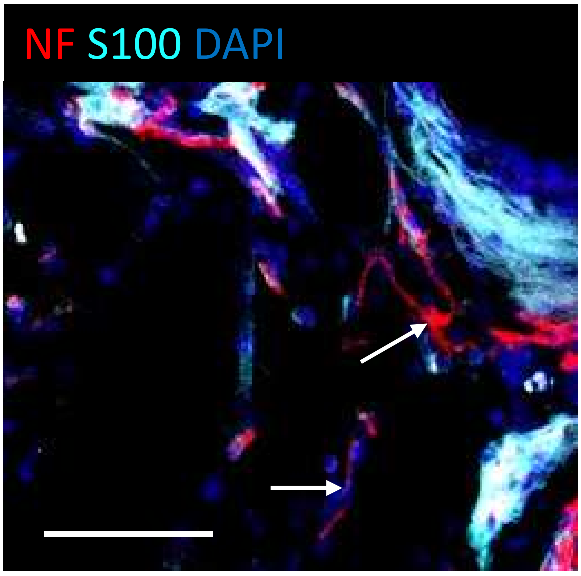

Legend Supplementary Fig. 3: Axon-Schwann cell association in the mMS lumen. Many but not all (arrow) NF+ axon profiles in the mMS lumen were found in close association with S100-expressing Schwann cells. Scale bar: 50 µm.
